# High-Resolution L-Band TomoSAR Imaging on Forest Canopies with UAV Swarm to Detect Dielectric Constant Anomaly

**DOI:** 10.3390/s23198335

**Published:** 2023-10-09

**Authors:** Hsu-Yueh Chuang, Jean-Fu Kiang

**Affiliations:** Graduate Institute of Communication Engineering, National Taiwan University, Taipei 10617, Taiwan

**Keywords:** TomoSAR, high resolution, L-band, focusing function, forest model, dielectric constant, reflectivity function, unmanned aerial vehicle, wildfire prediction

## Abstract

A rigorous TomoSAR imaging procedure is proposed to acquire high-resolution L-band images of a forest in a local area of interest. A focusing function is derived to relate the backscattered signals to the reflectivity function of the forest canopies without resorting to calibration. A forest voxel model is compiled to simulate different tree species, with the dielectric constant modeled with the Maxwell-Garnett mixing formula. Five different inverse methods are applied on two forest scenarios under three signal-to-noise ratios in the simulations to validate the efficacy of the proposed procedure. The dielectric-constant profile of trees can be used to monitor the moisture content of the forest. The use of a swarm of unmanned aerial vehicles (UAVs) is feasible to carry out TomoSAR imaging over a specific area to pinpoint potential spots of wildfire hazards.

## 1. Introduction

The morphology of a forest, including canopy height, vertical structure and spatial distribution, provides useful information for ecological protection [1], biomass estimation [2], the monitoring of biodiversity [1] and the global carbon cycle [3]. More incidences of forest fires in recent years may be correlated to the trend of global warming [4]. Regular monitoring of forest morphology may help in predicting potential wildfire spots for emergency preparation.

Wildfire risk increases as the soil dehydrates [4] or more dry bushes pile up [5]. L-band and C-band radars have been used to estimate the moisture content in the leaves, branches and trunks of trees [6], by detecting their dielectric constants [7].

In [8], an area-based approach was proposed to estimate different forms of deadwood. Light detection and ranging (LiDAR) has been used to retrieve 3D data of the forest structure, surface fuel depth, coverage and canopy density [9]. Synthetic aperture radar tomography (TomoSAR) in L- and P-bands has been used to reconstruct the 3D forest morphology via volume backscattering [10].

The soil and trunks are dominant scatterers in the P-band, while small branches are dominant scatterers in the L-band [11]. The backscattered signals in the L-band were reported to have a more uniform distribution in elevation [12]. In [13], an airborne P- and L-band TomoSAR technique using a Capon estimator was proposed to reconstruct a tropical forest, and the results were validated with small-footprint LiDAR (SFL) data. In [1], an airborne L-band TomoSAR technique was proposed to retrieve the vertical profile of a forest. In [14], an airborne L-band TomoSAR technique using wavelets was applied to survey the forest morphology, and its performance in conjunction with inverse algorithms of Fourier beamforming, Capon and compressive sensing, respectively, was analyzed.

To acquire TomoSAR images with high fidelity, the baseline aperture, baseline spacing, number of tracks and elevation ambiguity must be well orchestrated [15]. A method of coordinating baselines at nonuniform and uniform spacings was proposed by judging the corresponding point spread function [16]. As nonuniform baseline spacings may yield poor elevation resolution, a baseline aperture interpolator was proposed [2] to suppress sidelobes of the point spread function.

Inverse algorithms used for TomoSAR imaging of the reflectivity profile in the target volume can be roughly categorized into model-based (parametric), model-free (nonparametric) and hybrid [14]. Model-based algorithms include multiple signal classification (MUSIC) and covariance matching estimator (COMET). Model-free algorithms include Fourier beamforming (FB) and Capon beamforming (CB) [17]. In [18], a model-free iterative adaptive approach was shown to deliver finer elevation resolution than Fourier beamforming and Capon beamforming.

Compressive sensing (CS) techniques have been proposed to enable sparse SAR imaging methods [19,20]. In [21], a sparse SAR imaging method based on $\ell_{2,1}$-norm regularization was proposed to reduce the ghost-target phenomenon caused by azimuth periodic block sampling. In [22], an incremental SAR imaging method was proposed to improve the image quality and computational efficiency by reducing the azimuth ambiguity. In [23], a weighted sparse reconstruction method was proposed for single-channel SAR imaging on maritime targets to mitigate the azimuth ambiguities and improve the resolution.

In [16,17,18], several TomoSAR imaging methods were developed in terms of a normalized impulse response [24,25]. In [16], the effects of track number and track configuration on the acquired forest images were studied. It was reported that at least seven tracks were required to suppress the sidelobe level of the impulse response below −6 dB, resulting in a vertical resolution of 6 m. In [11], a weighted covariance fitting-based iterative spectral estimator (WISE) was proposed to retrieve the canopy height models and the above-ground biomass, achieving finer resolution than Capon and weighted-CS methods could deliver. In [14], the pros and cons of Capon beamforming, Fourier beamforming and CS algorithm were compared. In [18], a robust iterative adaptive approach (RIAA) in conjunction with a weighted least-squares criterion was proposed to conduct TomoSAR imaging with a small aperture of 5–30 m.

A vivid tree model is useful to validate a TomoSAR imaging method with fine spatial resolution. In [12,26], a forest was modeled as a reflectivity profile composed of a ground layer and a canopy layer. Three-dimensional tree generator software such as Arbaro (1995) [27], OnyxTREE (2020) [28] and AMAPstudio (2014) [29] were commonly used in LiDAR researches [30]. Arbaro is a rule-based growth algorithm [31], with which the trunks, branches and leaves of trees are transformed to a voxel model. A voxel model with a resolution of the order of a decimeter will be useful for pinpointing potential spots of wildfire hazards.

In this work, a rigorous L-band TomoSAR imaging method is proposed to reconstruct the dielectric-constant profile of a forest. A focusing function is derived to relate the backscattered signals to the reflectivity function along an elevation line segment at a specific azimuth and depth, without resorting to calibration. The profile of the reflectivity function along the elevation line segment is reconstructed with the inverse methods of compressive sensing (CS), Fourier beamforming (FB), multiple signal classification (MUSIC), amplitude and phase estimation (APES), and Capon, respectively. The simulated TomoSAR images acquired with these five inverse methods are analyzed in different scenarios under different signal-to-noise ratios.

SAR imaging tasks carried out on an unmanned aerial vehicle (UAV) were reported to achieve a fine spatial resolution of 10 cm [32]. The UAV navigation and trajectory for TomoSAR imaging has been studied [33]. A swarm of UAVs can be flexibly deployed to acquire high-resolution TomoSAR images of forest canopies, with the proposed TomoSAR imaging method.

The rest of this work is organized as follows. The forward problem and signal model are presented in Section 2, the TomoSAR imaging methods are presented in Section 3, the simulation results of acquired images are analyzed in Section 4 and some conclusions are drawn in Section 5.

## 2. Forward Problem and Signal Model

**Figure 1:**
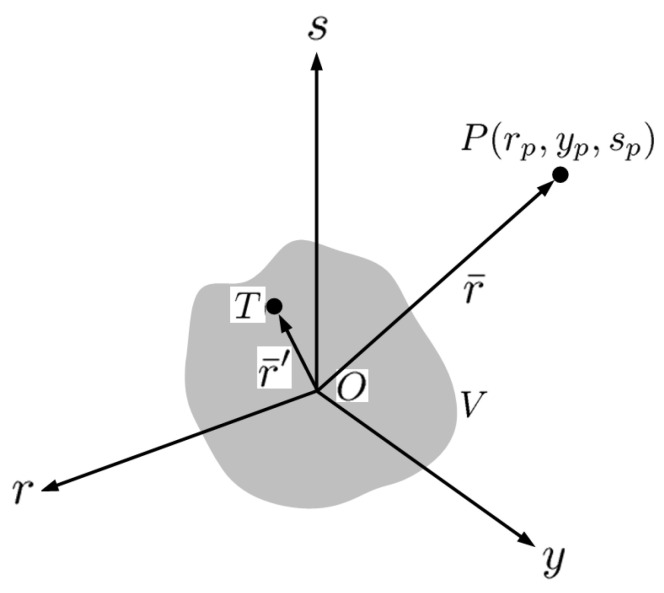
Scattering of electromagnetic wave by dielectric object in *V* [34], *P* at $\bar{r}=\hat{r}r_{p}+\hat{y}y_{p}+\hat{s}s_{p}$ is the observation point, and *T* at ${\bar{r}}^{\prime }=\hat{r}r^{\prime }+\hat{y}y+\hat{s}s$ denotes a point in volume *V*.

Figure 1 shows the schematic of electromagnetic scattering by a dielectric object in *V* with distribution of the refraction index $n({\bar{r}}^{\prime })$. An incident field $U_{i}\left(\bar{r},\tau \right)$ upon the object induces a scattered field $U_{s}\left(\bar{r},\tau \right)$, resulting in the total field $U(\bar{r},\tau )$. The origin is arbitrarily located within the volume *V*, the observation point *P* is given by $\bar{r}=\hat{r}r_{p}+\hat{y}y_{p}+\hat{s}s_{p}$ and a point in volume *V* is given by ${\bar{r}}^{\prime }=\hat{r}r^{\prime }+\hat{y}y+\hat{s}s$. The (r,y,s) coordinate system is related to the conventional (x,y,z) coordinate system by a rotation about the *y*-axis.

By taking the first-order Born approximation that $U\left(\bar{r},\tau \right)≃U_{i}\left(\bar{r},\tau \right)$ and the weak scattering assumption that $|U_{s}\left(\bar{r},\tau \right)\left|\ll \right|U_{i}\left(\bar{r},\tau \right)|$, the scattered field can be represented as [34]

(1) $$\begin{array}{ccc} & & \;U_{s}\left(\bar{r},\tau \right)=\frac{1}{4\pi }\int_{V}\frac{1-n^{2}\left({\bar{r}}^{\prime }\right)}{|\bar{r}-{\bar{r}}^{\prime }|c^{2}}\frac{\partial^{2}U_{i}\left({\bar{r}}^{\prime },\tau^{\prime }\right)}{\partial \tau^{\prime 2}}d{\bar{r}}^{\prime } \end{array}$$

where $\tau^{\prime }=\tau -\left|\bar{r}-{\bar{r}}^{\prime }\right|/c$.

Linear frequency modulation (LFM) pulses are periodically emitted from the radar to the object, with the waveform of $P\left(\tau \right)=\mathrm{rect}\left(\tau /T_{r}\right)e^{j(2\pi f_{0}\tau +\pi K_{r}\tau^{2})}$, where $T_{r}$ is the pulse duration, and $\mathrm{rect}(\tau /T_{r})$ is a window function of duration $T_{r}$. Thus, the incident field can be represented as

$$\begin{array}{ccc} & & \;U_{i}\left(\bar{r},\tau \right)=\frac{1}{4\pi }\frac{P\left(\tau -|\bar{r}-{\bar{r}}^{\prime }|/c\right)}{|\bar{r}-{\bar{r}}^{\prime }|} \end{array}$$

and the scattered field is reduced to

(2) $$\begin{array}{ccc} & & \;U_{s}\left(\bar{r},\tau \right)=\int_{V}\gamma \left({\bar{r}}^{\prime }\right)P\left(\zeta \right)d{\bar{r}}^{\prime } \end{array}$$

where $\zeta =\tau -2|\bar{r}-{\bar{r}}^{\prime }|/c$,

(3) $$\begin{array}{ccc} & & \;\gamma \left({\bar{r}}^{\prime }\right)=\frac{f_{0}^{\prime 2}\left[n^{2}\left({\bar{r}}^{\prime }\right)-1\right]}{4c^{2}{\left|\bar{r}-{\bar{r}}^{\prime }\right|}^{2}} \end{array}$$

is the reflectivity function [34], and $f_{0}^{\prime }=f_{0}+K_{r}\left(\tau^{\prime }-\left|\bar{r}-{\bar{r}}^{\prime }\right|/c\right)$, with $\tau^{\prime }=\tau -\left|\bar{r}-{\bar{r}}^{\prime }\right|/c$.

### 2.1. Range Compression

The backscattered signal in (2) is first demodulated to obtain the baseband signal $U_{b}\left(\bar{r},\tau \right)=U_{s}\left(\bar{r},\tau \right)e^{-j2\pi f_{0}\tau }$. A matched filter $P_{\mathrm{mf}}\left(u\right)=\mathrm{rect}\left(u/T_{r}\right)e^{-j\pi K_{r}u^{2}}$ [35] is then convolved with the baseband received signal $U_{b}\left(\tau ,\bar{r}\right)$ to give [34]

(4) $$\begin{array}{ccc} & & \;\int_{U}P_{\mathrm{mf}}\left(u\right)U_{b}\left(\bar{r},\tau -u\right)du=\int_{V}\gamma \left({\bar{r}}^{\prime }\right)Q_{b}\left(\right|\bar{r}-{\bar{r}}^{\prime }\left|,\tau \right)d{\bar{r}}^{\prime } \end{array}$$

where $Q_{b}\left(\right|\bar{r}-{\bar{r}}^{\prime }\left|,\tau \right)=Q_{r}\left(\right|\bar{r}-{\bar{r}}^{\prime }\left|,\tau \right)e^{-j4\pi f_{0}\left|\bar{r}-{\bar{r}}^{\prime }\right|/c}$ [35] and

(5) $$\begin{array}{ccc} & & \;Q_{r}\left(\right|\bar{r}-{\bar{r}}^{\prime }\left|,\tau \right)=-2\mathrm{sgn}\left(\zeta \right)\frac{\sin(2\pi K_{r}\zeta \left[\zeta -\mathrm{sgn}\left(\zeta \right)T_{r}\right]/2)}{2\pi K_{r}\zeta } \end{array}$$

is called the range factor [34].

### 2.2. Azimuth-Depth Compression

**Figure 2:**
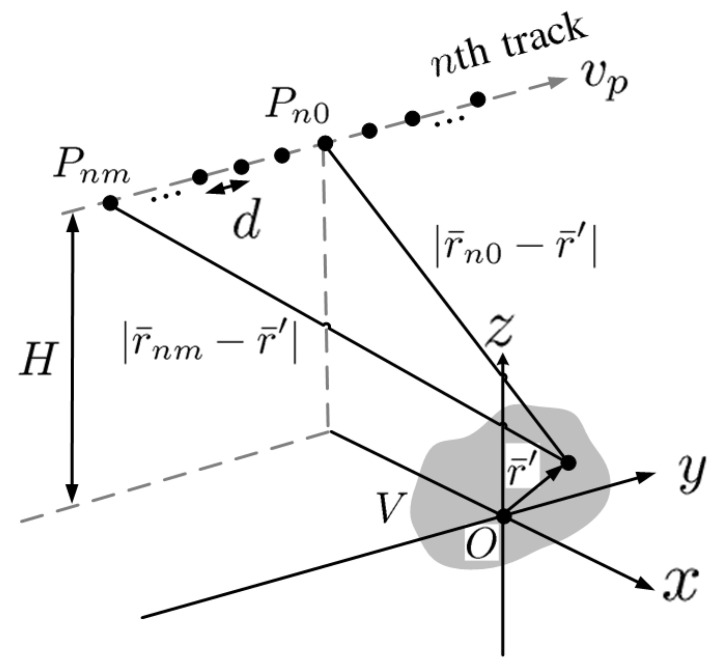
Schematic of azimuth sampling along track *n*, ${\bar{r}}_{nm}$ is the platform position at the *m*th sampling time, *d* is azimuth spacing.

Figure 2 shows the schematic of azimuth sampling along track *n*. The sampling position $P_{nm}$ is located at $\bar{r}={\bar{r}}_{nm}$, with $-N_{a}/2\leq m\leq N_{a}/2$ and uniform spacing *d*. The range between $P_{nm}$ and a target point at ${\bar{r}}^{\prime }$ can be approximated as

(6) $$\begin{array}{ccc} & & |{\bar{r}}_{nm}-{\bar{r}}^{\prime }\left|≃\right|{\bar{r}}_{n0}-{\bar{r}}^{\prime }|+\frac{{\left(md\right)}^{2}}{2|{\bar{r}}_{n0}|} \end{array}$$

Thus, the phase term in (4) is approximated as

(7) $$\begin{array}{ccc} & & \;e^{-j4\pi f_{0}\left|{\bar{r}}_{nm}-{\bar{r}}^{\prime }\right|/c}≃e^{-j4\pi f_{0}\left|{\bar{r}}_{n0}-{\bar{r}}^{\prime }\right|/c}\Psi_{nm}\left({\bar{r}}^{\prime }\right) \end{array}$$

where $\Psi_{nm}\left({\bar{r}}^{\prime }\right)=e^{-j2\pi m^{2}d^{2}/\left(\lambda \right|{\bar{r}}_{n0}\left|\right)}$.

Let us define an azimuth reference function $\Phi_{n(k-m)}=e^{j2\pi {\left(k-m\right)}^{2}d^{2}/\left(\lambda \right|{\bar{r}}_{n0}\left|\right)}$, then multiply it to both sides of (4) and sum over $-N_{a}/2\leq m\leq N_{a}/2$ to give

(8) $$\begin{array}{ccc} & & \;\sum_{m=-N_{a}/2}^{N_{a}/2}\int_{U}P_{\mathrm{mf}}\left(u\right)U_{b}\left({\bar{r}}_{nm},\tau -u\right)du\Phi_{n(k-m)}\\ & & \;=\int_{V}\gamma \left({\bar{r}}^{\prime }\right)\sum_{m=-N_{a}/2}^{N_{a}/2}Q_{b}\left(\right|{\bar{r}}_{nm}-{\bar{r}}^{\prime }\left|,\tau \right)\Phi_{n(k-m)}d{\bar{r}}^{\prime }\\ & & \;=\int_{V}\gamma \left({\bar{r}}^{\prime }\right)e^{-j4\pi f_{0}\left|{\bar{r}}_{n0}-{\bar{r}}^{\prime }\right|/c}\xi_{nk}\left(\tau ,{\bar{r}}^{\prime }\right) \end{array}$$

where

(9) $$\begin{array}{ccc} & & \;\xi_{nk}\left(\tau ,{\bar{r}}^{\prime }\right)=\sum_{m=-N_{a}/2}^{N_{a}/2}Q_{r}\left(\right|{\bar{r}}_{nm}-{\bar{r}}^{\prime }\left|,\tau \right)\Psi_{nm}\left({\bar{r}}^{\prime }\right)\Phi_{n(k-m)}\\ & & \;=-\sum_{m=-N_{a}/2}^{N_{a}/2}\mathrm{sgn}\left(\zeta_{nm}\right)\left[\zeta_{nm}-\mathrm{sgn}\left(\zeta_{nm}\right)T_{r}\right]\\ & & \mathrm{sinc}\left(K_{r}\zeta_{nm}\left[\zeta_{nm}-\mathrm{sgn}\left(\zeta_{nm}\right)T_{r}\right]\right)e^{-j2\pi m^{2}d^{2}/\left(\lambda \right|{\bar{r}}_{n0}\left|\right)}e^{j2\pi {\left(k-m\right)}^{2}d^{2}/\left(\lambda \right|{\bar{r}}_{n0}\left|\right)} \end{array}$$

with sinc(x)=sin(πx)/(πx) and $\zeta_{nm}=\tau -2\left|{\bar{r}}_{nm}-{\bar{r}}^{\prime }\right|/c$. By making the approximation $\zeta_{nm}≃\zeta_{n0}$, (9) is further reduced to

(10) $$\begin{array}{ccc} & & \;\xi_{nk}\left(\tau ,{\bar{r}}^{\prime }\right)≃-\mathrm{sgn}\left(\zeta_{n0}\right)\left[\zeta_{n0}-\mathrm{sgn}\left(\zeta_{n0}\right)T_{r}\right]e^{j2\pi k^{2}d^{2}/\left(\lambda \right|{\bar{r}}_{n0}\left|\right)}\\ & & \mathrm{sinc}\left(K_{r}\zeta_{n0}\left[\zeta_{n0}-\mathrm{sgn}\left(\zeta_{n0}\right)T_{r}\right]\right)\frac{\sin[\left(N_{a}+1\right)2\pi kd^{2}/\left(\lambda \right|{\bar{r}}_{n0}\left|)\right]}{\sin[2\pi kd^{2}/\left(\lambda \right|{\bar{r}}_{n0}\left|)\right]} \end{array}$$

**Figure 3:**
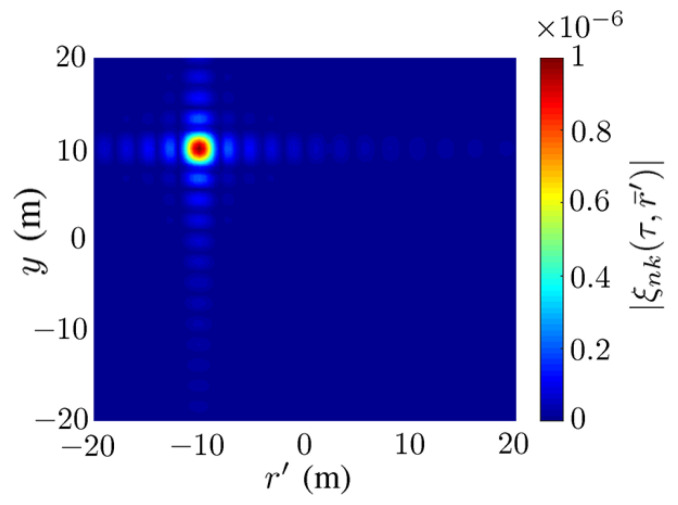
Magnitude of $\xi_{nk}\left(\tau ,{\bar{r}}^{\prime }\right)$ in (10), $D_{y}=0.5$ m and $D_{r}=0.5$ m, $\tau =2|{\bar{r}}_{n0}-{\bar{r}}^{\prime \prime }|/c$, k=20, $y^{\prime \prime }=10$ m, $r^{\prime \prime }=-10$ m.

Figure 3 shows the amplitude of $\xi_{nk}\left(\tau ,{\bar{r}}^{\prime }\right)$ in (10), with −20≤y≤20 and $-20\leq r^{\prime }\leq 20$. The magnitude of $\xi_{nk}\left(\tau ,{\bar{r}}^{\prime }\right)$ manifests a conspicuous peak at $r^{\prime \prime }=-10$ m and $y^{\prime \prime }=10$ m (k=20), similar to a conventional point spread function or a delta function.

Since $\xi_{nk}\left(\tau ,{\bar{r}}^{\prime }\right)$ is concentrated about $r^{\prime \prime }=r^{\prime }$, (8) is further approximated as

(11) $$\begin{array}{ccc} & & \;\sum_{m=-N_{a}/2}^{N_{a}/2}\int_{U}P_{\mathrm{mf}}\left(u\right)U_{b}\left({\bar{r}}_{nm},\tau -u\right)du\Phi_{n(k-m)}\\ & & \;≃D_{r}D_{y}\int_{S_{nk}\left(r^{\prime \prime }\right)}\gamma \left({\bar{r}}^{\prime }\right)e^{-j4\pi f_{0}\left|{\bar{r}}_{n0}-{\bar{r}}^{\prime }\right|/c}\xi_{nk}\left(\tau ,{\bar{r}}^{\prime }\right)ds_{n} \end{array}$$

where $D_{r}$ and $D_{y}$ are the spatial resolutions in range (*r*) and azimuth (*y*), respectively, $S_{nk}\left(r^{\prime \prime }\right)$ denotes a sequence of voxels along a line parallel to the *s* axis viewed from track *n*, with $r^{\prime \prime }=r^{\prime }$ and $y=kD_{y}$.

## 3. TomoSAR Imaging Methods

**Figure 4:**
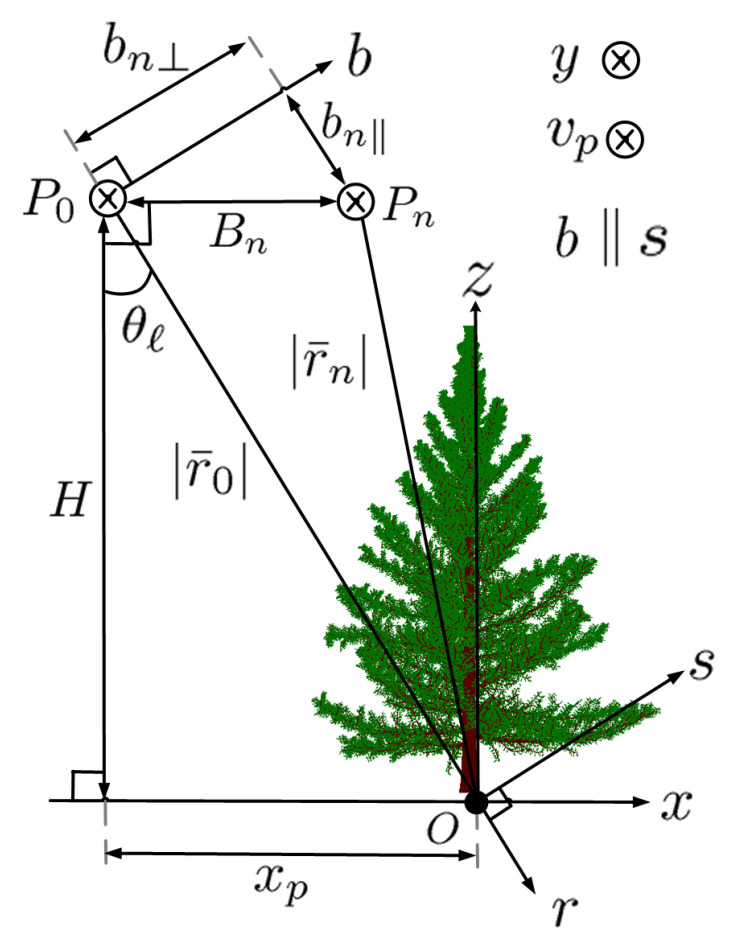
Schematic of TomoSAR imaging with platforms $P_{0}$ and $P_{n}$ along master track and track *n*, respectively [14], which are separated by perpendicular baseline $b_{n⊥}$, parallel baseline $b_{n\|}$ and parallel distance $B_{n}$.

Figure 4 shows the schematic of TomoSAR imaging. Without loss of generality, all the tracks are assumed to be at the same height *H*. The side-looking angle from platform $P_{0}$ along the master track at η=0 to point *O* is $\theta_{\ell }$. The *b* axis is perpendicular to $\bar{P_{0}O}$ and passes through $P_{0}$. The *s* axis is parallel to the *b* axis and passes through *O*.

The backscattered signal from the *q*th voxel is received along the *n*th track and demodulated to the baseband as

(12) $$\begin{array}{ccc} & & \;u_{1nq}\left(\tau ,\eta_{m}\right)=\Delta V_{q}\gamma \left({\bar{r}}_{q}^{\prime }\right)\mathrm{rect}\left(\frac{\tau -2|{\bar{r}}_{nm}-{\bar{r}}_{q}^{\prime }|/c}{T_{r}}\right)\\ & & e^{-j4\pi f_{0}|{\bar{r}}_{nm}-{\bar{r}}_{q}^{\prime }|/c+j\pi K_{r}\left(\tau -2\right|{\bar{r}}_{nm}-{\bar{r}}_{q}^{\prime }{\left|/c\right)}^{2}} \end{array}$$

where $\Delta V_{q}$ is the volume of the *q*th voxel, and $\eta_{m}=md/v_{p}$. The received baseband signals from all the *Q* voxels are given by

(13) $$\begin{array}{ccc} & & \;U_{s}\left(\tau ,\eta_{m}\right)=\Delta V\sum_{q=1}^{Q}\gamma \left({\bar{r}}_{q}^{\prime }\right)\mathrm{rect}\left(\frac{\tau -2|{\bar{r}}_{nm}-{\bar{r}}_{q}^{\prime }|/c}{T_{r}}\right)\\ & & e^{j2\pi f_{0}\left(\tau -2\right|{\bar{r}}_{nm}-{\bar{r}}_{q}^{\prime }|/c)+j\pi K_{r}\left(\tau -2\right|{\bar{r}}_{nm}-{\bar{r}}_{q}^{\prime }{\left|/c\right)}^{2}} \end{array}$$

By applying range compression and azimuth-depth compression as prescribed in the last Section, we have

(14) $$\begin{array}{ccc} & & \;g_{nk}\left(\tau_{n}\left(r^{\prime \prime }\right)\right)=\sum_{m=-N_{a}/2}^{N_{a}/2}\int_{U}P_{\mathrm{mf}}\left(u\right)U_{b}\left({\bar{r}}_{nm},\tau -u\right)du\Phi_{n(k-m)}\\ & & \;≃D_{r}D_{y}\int_{S_{nk}\left(r^{\prime \prime }\right)}\gamma \left({\bar{r}}^{\prime }\right)e^{-j4\pi f_{0}\left|{\bar{r}}_{n0}-{\bar{r}}_{n}^{\prime }\right|/c}\xi_{nk}\left(\tau_{n}\left(r^{\prime \prime }\right),{\bar{r}}_{n}^{\prime }\right)ds_{n} \end{array}$$

which is the acquired single-look complex (SLC) image along track *n* at y=kd, where (11) is used and

(15) $$\begin{array}{ccc} & & \;\tau_{n}\left(r^{\prime \prime }\right)=2\left|{\bar{r}}_{n0}-{\bar{r}}^{\prime \prime }\right|/c \end{array}$$

is the round-trip time between ${\bar{r}}_{n0}$ and ${\bar{r}}^{\prime \prime }$.

In conventional TomoSAR imaging methods, the original 3D inverse problem is decomposed into multiple inverse problems of solving a 1D SLC image as [24,25]

$$\begin{array}{ccc} & & \;g_{n}=\int_{s_{\min}}^{s_{\max}}\gamma \left(s\right)e^{j\phi (s)}ds \end{array}$$

where $g_{n}$ is the SLC image along track *n*, *s* is the coordinate along an elevation line segment at specific azimuth *y* and depth $r^{\prime \prime }$, γ(s) is the reflectivity function to be reconstructed and ϕ(s) is a phase function pertinent to track location and coordinate *s*.

In [24], a spectral estimation for coherent adaptive nulling (SPECAN) algorithm was proposed to acquire SAR images at medium-to-low resolution. The received signal was modeled as a convolution of reflectivity function and a deramped phase, usually involving some calibration or normalization. In [25], a 2D point spread function (PSF) was proposed, which was later approximated as a 2D Dirac delta function to simplify the formulation. The SLC image acquired along track *n* was represented as a convolution of the 2D PSF and the reflectivity function multiplied with a phase function accounting for the round-trip range between the observation point and target point.

In our procedure, a rigorous scattering theory is used to derive the backscattered signals from the target volume. A focusing function at specific azimuth *y* and depth $r^{\prime \prime }$ is derived to relate the backscattered signals to the reflectivity function, without resorting to calibration or normalization. The focusing function, $\xi_{nk}\left(\tau_{n}\left(r^{\prime \prime }\right),{\bar{r}}^{\prime }\right)$, manifests a similar feature of a point spread function in the azimuth-depth plane, as in [25].

### 3.1. Covariance of Deramped Images

Next, multiply $g_{nk}\left(\tau_{n}\left(r^{\prime \prime }\right)\right)$ with $e^{j4\pi f_{0}\left|{\bar{r}}_{n0}\right|/c}$ to derive a deramped image ${\tilde{g}}_{nk}\left(\tau_{n}\left(r^{\prime \prime }\right)\right)$, then compute the covariance between the two SLC deramped images along tracks *n* and $n^{\prime }$, respectively, as [36]

(16) $$\begin{array}{ccc} & & \;R_{knn^{\prime }}\left(r^{\prime \prime }\right)=E\{{\tilde{g}}_{nk}\left(\tau_{n}\left(r^{\prime \prime }\right)\right){\tilde{g}}_{n^{\prime }k}^{*}\left(\tau_{n^{\prime }}\left(r^{\prime \prime }\right)\right)\\ & & \;=D_{r}^{2}D_{y}^{2}\int_{S_{nk}\left(r^{\prime \prime }\right)}ds_{n}\int_{S_{n^{\prime }k}\left(r^{\prime \prime }\right)}ds_{n^{\prime }}E\left\{\gamma \left({\bar{r}}_{n}^{\prime }\right)\gamma^{*}\left({\bar{r}}_{n^{\prime }}^{\prime }\right)\right\}\\ & & e^{-j4\pi f_{0}\left(\right|{\bar{r}}_{n0}-{\bar{r}}_{n}^{\prime }\left|-\right|{\bar{r}}_{n0}\left|\right)/c}e^{j4\pi f_{0}\left(\right|{\bar{r}}_{n^{\prime }0}-{\bar{r}}_{n^{\prime }}^{\prime }\left|-\right|{\bar{r}}_{n^{\prime }0}\left|\right)/c}\xi_{nk}\left(\tau_{n}\left(r^{\prime \prime }\right),{\bar{r}}_{n}^{\prime }\right)\xi_{n^{\prime }k}^{*}\left(\tau_{n^{\prime }}\left(r^{\prime \prime }\right),{\bar{r}}_{n^{\prime }}^{\prime }\right) \end{array}$$

where ${\bar{r}}_{n}^{\prime }\in S_{nk}\left(r^{\prime \prime }\right)$ and ${\bar{r}}_{n^{\prime }}^{\prime }\in S_{n^{\prime }k}\left(r^{\prime \prime }\right)$.

Then, the autocorrelation of the reflectivity function is approximated as [36]

(17) $$\begin{array}{ccc} & & \;E\left\{\gamma \left({\bar{r}}_{n}^{\prime }\right)\gamma^{*}\left({\bar{r}}_{n^{\prime }}^{\prime }\right)\right\}≃{\left|\gamma \left({\bar{r}}_{n}^{\prime }\right)\right|}^{2}\delta \left({\bar{r}}_{n}^{\prime }-{\bar{r}}_{n^{\prime }}^{\prime }\right) \end{array}$$

where $\delta ({\bar{r}}_{n}^{\prime }-{\bar{r}}_{n^{\prime }}^{\prime })$ is the Dirac delta function. In practice, $S_{nk}\left(r^{\prime \prime }\right)$ and $S_{n^{\prime }k}\left(r^{\prime \prime }\right)$ almost overlap with each other, and the difference between the look angles, $\Delta \theta_{nn^{\prime }}=\theta_{n}-\theta_{n^{\prime }}$, cannot be ignored. Thus, (16) is reduced to [14,37]

(18) $$\begin{array}{ccc} & & \;R_{knn^{\prime }}\left(r^{\prime \prime }\right)=\int_{S_{nk}\left(r^{\prime \prime }\right)}F_{knn^{\prime }}\left(s_{n},r^{\prime \prime }\right)e^{-j\kappa_{nn^{\prime }}s_{n}}ds_{n} \end{array}$$

where

(19) $$\begin{array}{ccc} & & \;F_{knn^{\prime }}\left(s_{n},r^{\prime \prime }\right)=D_{r}^{2}D_{y}^{2}{\left|\gamma \left({\bar{r}}_{n}^{\prime }\right)\right|}^{2}\xi_{nk}\left(\tau_{n}\left(r^{\prime \prime }\right),{\bar{r}}_{n}^{\prime }\right)\xi_{n^{\prime }k}^{*}\left(\tau_{n^{\prime }}\left(r^{\prime \prime }\right),{\bar{r}}_{n}^{\prime }\right) \end{array}$$

is called the vertical reflectivity profile, ${\bar{r}}_{n}^{\prime }=\hat{s}s_{n}+\hat{y}kd+\hat{r}r^{\prime \prime }$, and $\kappa_{nn^{\prime }}=4\pi \Delta \theta_{nn^{\prime }}/\lambda_{0}$ is the vertical wavenumber, with $0\leq n,n^{\prime }\leq N_{p}$.

The covariances between all possible pairs of SLC deramped images are compiled into a covariance matrix as [38]

$$\begin{array}{ccc} & & \;{\bar{\bar{R}}}_{k}\left(r^{\prime \prime }\right)=\left[\begin{array}{cc} E\{{\tilde{g}}_{0k}\left(\tau_{0},r^{\prime \prime }\right){\tilde{g}}_{0k}^{*}\left(\tau_{0},r^{\prime \prime }\right)\} & \cdots \\ \vdots & \ddots \\ E\{{\tilde{g}}_{N_{p}k}\left(\tau_{N_{p}},r^{\prime \prime }\right){\tilde{g}}_{0k}^{*}\left(\tau_{0},r^{\prime \prime }\right)\} & \cdots \end{array}\right.\\ & & {\left.\begin{array}{cc} \cdots & E\{{\tilde{g}}_{0k}\left(\tau_{0},r^{\prime \prime }\right){\tilde{g}}_{N_{p}k}^{*}\left(\tau_{N_{p}},r^{\prime \prime }\right)\}\\ \ddots & \vdots \\ \cdots & E\{{\tilde{g}}_{N_{p}k}\left(\tau_{N_{p}},r^{\prime \prime }\right){\tilde{g}}_{N_{p}k}^{*}\left(\tau_{N_{p}},r^{\prime \prime }\right)\} \end{array}\right]}_{\left(N_{p}+1\right)\times \left(N_{p}+1\right)} \end{array}$$

which is vectorized into ${\left.{\bar{R}}_{k}\left(r^{\prime \prime }\right)\right|}_{{\left(N_{p}+1\right)}^{2}\times 1}$ [14]. Similarly, all the vertical wavenumbers are compiled into a vertical wavenumber matrix as

(20) $$\begin{array}{ccc} & & \;\overset{=}{K}={\left[\begin{array}{cccc} \kappa_{00} & \kappa_{01} & \cdots & \kappa_{0N_{p}}\\ \kappa_{10} & \ddots & & \kappa_{1N_{p}}\\ \vdots & & \ddots & \vdots \\ \kappa_{N_{p}0} & \cdots & \cdots & \kappa_{N_{p}N_{p}} \end{array}\right]}_{\left(N_{p}+1\right)\times \left(N_{p}+1\right)} \end{array}$$

which is vectorized into ${\left.\bar{K}\right|}_{{\left(N_{p}+1\right)}^{2}\times 1}$.

The value of $F_{knn^{\prime }}\left(s_{n},r^{\prime \prime }\right)$ is insensitive to *n* or $n^{\prime }$; hence, we select a set of grid points (voxels) along a common *s* axis in a vector $\bar{s}={\left[s_{1},s_{2},\cdots ,s_{L}\right]}^{t}$, independent of *n* or $n^{\prime }$. The values of $F_{knn^{\prime }}\left(s_{n},r^{\prime \prime }\right)$ at the grid points $\left\{s_{n}\right\}$ are put in a vector ${\bar{F}}_{k}\left(r^{\prime \prime }\right)={\left[F_{k}\left(s_{1},r^{\prime \prime }\right),F_{k}\left(s_{2},r^{\prime \prime }\right),\cdots ,F_{k}\left(s_{L},r^{\prime \prime }\right)\right]}_{L\times 1}^{t}$, which is also independent of *n* or $n^{\prime }$. Define a steering matrix on the grid points $\left\{s_{n}\right\}$ as $\overset{=}{A}\left(\bar{s}\right)={\left[e^{-j\bar{K}s_{1}},e^{-j\bar{K}s_{2}},\cdots ,e^{-j\bar{K}s_{L}}\right]}_{{\left(N_{p}+1\right)}^{2}\times L}$ [14]. Thus, (18) is discretized into

(21) $$\begin{array}{ccc} & & \;{\bar{R}}_{k}\left(r^{\prime \prime }\right)=\overset{=}{A}\left(\bar{s}\right)\cdot {\bar{F}}_{k}\left(r^{\prime \prime }\right) \end{array}$$

The backscattered signals are related to the vertical reflectivity profile, $F_{knn}\left(s_{n},r^{\prime \prime }\right)$, which is the counterpart of γ(s) in [39] and σ(x,γ) in [19]. The focusing function proposed in this work is the counterpart of the point spread function or delta function in conventional TomoSAR imaging methods. Since the focusing function is derived from the scattering theory, no calibration or normalization on the reconstructed reflectivity function is required.

The main contribution of our work is to establish a rigorous formulation of backscattered signals in terms of the reflectivity function via the scattering theory, without resorting to the empirical point spread function or delta function. The focusing function manifests a similar feature of PSF yet retains all of the original information. After range compression, azimuth-depth compression, co-registration and covariance among SLC images, an inverse problem in (21) is formed, which is solved by applying various inverse methods, including compressive sensing [40], Fourier beamforming [14,37], multiple signal classification (MUSIC) [19], amplitude and phase estimation (APES) [41] and Capon [14]. The acquired distributions of the dielectric constant in the target volume is analyzed to validate our procedure and compare the pros and cons of different inverse methods.

### 3.2. Compressive Sensing Method

The unknowns ${\bar{F}}_{k}\left(r^{\prime \prime }\right)$ in (21) at *L* grid points along *s* direction are relabeled as $\bar{u}\left(\bar{s}\right)={\left[u_{1},u_{2},\cdots ,u_{L}\right]}^{t}$. Equation (21) is then solved with the compressive-sensing method as [40]

(22) $$\begin{array}{ccc} & & \;\tilde{\bar{u}}\left(\bar{s}\right)=\arg\;\min_{\bar{u}\left(\bar{s}\right)}{∥\bar{u}\left(\bar{s}\right)∥}_{1}\;\;s.t.\;\;{\bar{R}}_{k}\left(r^{\prime \prime }\right)=\bar{\bar{A}}\left(\bar{s}\right)\cdot \bar{u}\left(\bar{s}\right) \end{array}$$

where $\bar{\bar{A}}\left(\bar{s}\right)$ plays the role of a dictionary. By substituting $F_{knn^{\prime }}\left(s_{n},r^{\prime \prime }\right)$ in (19) with $\tilde{\bar{u}}\left(\bar{s}\right)$, the reflectivity $\left|\gamma ({\bar{r}}_{n}^{\prime })\right|$ is obtained without resorting to calibration or normalization. Then, the dielectric constant can be estimated by using (3).

Equation (22) is equivalent to [14]

(23) $$\begin{array}{ccc} & & \;\tilde{\bar{u}}\left(\bar{s}\right)=\arg\min_{\bar{u}\left(\bar{s}\right)}\left\{{∥{\bar{R}}_{k}\left(r^{\prime \prime }\right)-\bar{\bar{A}}\left(\bar{s}\right)\cdot \bar{u}\left(\bar{s}\right)∥}_{2}^{2}+\lambda_{s}{∥\bar{u}\left(\bar{s}\right)∥}_{1}\right\} \end{array}$$

where $∥\bar{f}{∥}_{1}$ and $∥\bar{f}{∥}_{2}$ are $L_{1}$-norm and $L_{2}$-norm, respectively, of $\bar{f}$, and $\lambda_{s}$ is a hyperparameter [40]. The CVX tool can be applied to solve (22) or (23). The reconstructed reflectivity profile is sensitive to the chosen value of $\lambda_{s}$, which is varied between 0.1 and 1. Hence, (22) is adopted in this work.

### 3.3. Fourier Beamforming (FB) Method

The SLC images ${\tilde{g}}_{nk}\left(\tau_{n}\left(r^{\prime \prime }\right)\right)$’s acquired along all the tracks are co-registered at y=kd and $r=R_{0}+r^{\prime \prime }$ to constitute a measurement vector

(24) $$\begin{array}{ccc} & & \;\bar{g}={\left[\begin{array}{c} {\tilde{g}}_{0k}\left(\tau_{0}\left(r^{\prime \prime }\right)\right)\\ {\tilde{g}}_{1k}\left(\tau_{0}\left(r^{\prime \prime }\right)\right)\\ \vdots \\ {\tilde{g}}_{N_{p}k}\left(\tau_{0}\left(r^{\prime \prime }\right)\right) \end{array}\right]}_{(N_{p}+1)\times 1} \end{array}$$

where $\tau_{0}\left(r^{\prime \prime }\right)$ is the round-trip time between the master track and ${\bar{r}}^{\prime \prime }$, as defined in (15). The phase terms in (14) along all the tracks constitute a steering vector as

(25) $$\begin{array}{ccc} & & \;\bar{a}\left(s\right)={\left[\begin{array}{c} e^{j2k_{0}b_{0}s/\left(R_{0}+r^{\prime \prime }\right)}\\ e^{j2k_{0}b_{1}s/\left(R_{0}+r^{\prime \prime }\right)}\\ \vdots \\ e^{j2k_{0}b_{N_{p}}s/\left(R_{0}+r^{\prime \prime }\right)} \end{array}\right]}_{(N_{p}+1)\times 1} \end{array}$$

Without loss of generality, the baseline of the master track is set to $b_{0}=0$. The steering vectors at all the grid points along the *s* axis constitute a steering matrix as

$$\begin{array}{ccc} & & \;\bar{\bar{A}}={\left[\bar{a}\left(s_{1}\right),\bar{a}\left(s_{2}\right),\cdots ,\bar{a}\left(s_{L}\right)\right]}_{(N_{p}+1)\times L} \end{array}$$

By using (24) and (25), the signal model is rephrased as

(26) $$\begin{array}{ccc} & & \;\bar{g}=\bar{\bar{A}}\left(s\right)\cdot \bar{u}\left(s\right) \end{array}$$

where $\bar{u}\left(s\right)={\left[u_{1},u_{2},\cdots ,u_{L}\right]}^{t}$ is the vertical reflectivity profile sampled at *L* grid points along the *s* axis. The image acquired with the FB method is [37]

$$\begin{array}{ccc} & & \;{\bar{u}}_{F}\left(s\right)=\frac{1}{{\left(N_{p}+1\right)}^{2}}{\bar{h}}_{F}^{†}\left(s\right)\cdot \bar{\bar{R}}\cdot {\bar{h}}_{F}\left(s\right) \end{array}$$

where

$$\begin{array}{ccc} & & \;\bar{\bar{R}}=\frac{1}{N_{p}+1}\sum_{n=1}^{N_{p}+1}{\bar{g}}_{n}{\bar{g}}_{n}^{†} \end{array}$$

is the covariance matrix of SLC images and

$$\begin{array}{ccc} & & \;{\bar{h}}_{F}\left(s\right)=\frac{\bar{a}\left(s\right)}{{\bar{a}}^{†}\left(s\right)\cdot \bar{a}\left(s\right)} \end{array}$$

### 3.4. Multiple Signal Classification (MUSIC) Method

The covariance matrix $\bar{\bar{R}}$ among SLC images can be decomposed as $\bar{\bar{R}}=\bar{\bar{U}}\cdot \bar{\bar{D}}\cdot {\bar{\bar{U}}}^{†}$, where $\bar{\bar{D}}$ is the eigenvalue matrix sorted in descending order, namely, $\lambda_{1}\geq \lambda_{2}\geq \cdots \geq \lambda_{N_{p}-1}\geq \lambda_{N_{p}}$. The eigenvectors corresponding to the largest *K* eigenvalues are compiled into a signal matrix $\bar{\bar{S}}$, and the remaining $N_{p}-K$ eigenvectors are compiled into a noise matrix $\bar{\bar{N}}$, where the choice of *K* is contingent upon the SLC images. The image acquired with the MUSIC method is [37]

$$\begin{array}{ccc} & & \;{\bar{u}}_{M}\left(s\right)=\frac{1}{\bar{a}\left(s\right)\cdot \bar{\bar{N}}\cdot {\bar{\bar{N}}}^{†}\cdot {\bar{a}}^{†}\left(s\right)} \end{array}$$

Empirical parameter *K* is related to the actual number of phase centers in the complex forest structures. For the proposed simulation scenario, *K* is varied from 1 to 5 and the resulting images suggest that K=2 is the most suitable choice. It is observed that as *K* increases, the reconstructed image tends to display ghost targets.

### 3.5. Amplitude and Phase Estimation (APES) Method

The amplitude and phase estimation (APES) method is implemented by optimizing a filter with finite impulse response (FIR)

(27) $$\begin{array}{ccc} & & \;\bar{h}={\left[h_{1},h_{2},\cdots ,h_{N_{p}+1}\right]}^{†} \end{array}$$

which is applied to the SLC image $\bar{g}$ in (24) to obtain an output

(28) $$\begin{array}{ccc} & & \;y=\sum_{n=1}^{N_{p}+1}h\left[n\right]g\left[n\right]={\bar{h}}^{†}\cdot \bar{g} \end{array}$$

of which the power is given by

(29) $$\begin{array}{ccc} & & {\;E\{|y|}^{2}\}=E\{|{\bar{h}}^{†}\cdot \bar{g}{|}^{2}\}=E\left\{{\bar{h}}^{†}\cdot \bar{g}{\bar{g}}^{†}\cdot \bar{h}\right\}={\bar{h}}^{†}\cdot \bar{\bar{R}}\cdot \bar{h} \end{array}$$

where $\bar{\bar{R}}=E\left\{\bar{g}{\bar{g}}^{†}\right\}$ is the covariance matrix of SLC images. By substituting (26) into (28), we obtain

(30) $$\begin{array}{ccc} & & \;y={\bar{h}}^{†}\cdot \bar{g}={\bar{h}}^{†}\cdot \bar{\bar{A}}\cdot \bar{u}={\bar{h}}^{†}\cdot \bar{a}\left(s_{1}\right)u_{1}+{\bar{h}}^{†}\cdot \bar{a}\left(s_{2}\right)u_{2}+\cdots +{\bar{h}}^{†}\cdot \bar{a}\left(s_{L}\right)u_{L} \end{array}$$

The vertical reflectivity profile is acquired by solving the following optimization problem

(31) $$\begin{array}{ccc} & & \;\tilde{\bar{u}}=\arg\;\min_{\bar{u}}{\left|\bar{g}-\bar{\bar{A}}\cdot \bar{u}\right|}^{2} \end{array}$$

If the filter $\bar{h}$ is designed to retrieve specific $u_{\ell }$ at $s_{\ell }$ while suppressing the other *u* components, namely,

(32) $$\begin{array}{ccc} & & \;{\bar{h}}^{†}\cdot \bar{a}\left(s_{\ell }\right)=1 \end{array}$$

(33) $$\begin{array}{ccc} & & \;{\bar{h}}^{†}\cdot \left[\bar{a}\left(s_{1}\right)u_{1}+\cdots +\bar{a}\left(s_{\ell -1}\right)u_{\ell -1}+\bar{a}\left(s_{\ell +1}\right)u_{\ell +1}+\cdots +\bar{a}\left(s_{L}\right)u_{L}\right]\to 0 \end{array}$$

then (31) is reduced to an optimization problem on $\bar{h}$ as [37]

(34) $$\begin{array}{ccc} & & \;{\bar{h}}_{A}\left(s\right)=\min_{\bar{h}}\;{\bar{h}}^{†}\cdot \bar{\bar{R}}\cdot \bar{h}\;\;s.t.\;\;{\bar{h}}^{†}\cdot \bar{a}\left(s\right)=1 \end{array}$$

where $s_{\ell }$ is relabeled as *s*. The optimal solution of (34) is given by [37]

(35) $$\begin{array}{ccc} & & \;{\bar{h}}_{A}\left(s\right)=\frac{{\left(\bar{\bar{R}}+\lambda \bar{\bar{I}}\right)}^{-1}\cdot \bar{a}\left(s\right)}{{\bar{a}}^{†}\left(s\right)\cdot {\left(\bar{\bar{R}}+\lambda \bar{\bar{I}}\right)}^{-1}\cdot \bar{a}\left(s\right)} \end{array}$$

where λ>0 is an empirical parameter used to avoid possible singularity in matrix inversion [37]. Next, the optimal solution of u(s) is determined as

(36) $$\begin{array}{ccc} & & \;{\bar{u}}_{A}\left(s\right)={\bar{h}}_{A}^{†}\left(s\right)\cdot \bar{g} \end{array}$$

then the reflectivity function is estimated as

(37) $$\begin{array}{ccc} & & |\tilde{\gamma }\left(s\right)|≃\frac{1}{D_{r}D_{y}}\sqrt{\frac{|{\bar{u}}_{A}\left(s\right)|}{|\xi_{0k}\left(\tau_{0}\left(r^{\prime \prime }\right),{\bar{r}}^{\prime }\right)\xi_{0k}^{*}\left(\tau_{0}\left(r^{\prime \prime }\right),{\bar{r}}^{\prime }\right)|}} \end{array}$$

under the approximation that $u_{A}\left(s\right)$ is insensitive to *n* or $n^{\prime }$. By using (3), the dielectric constant is estimated as

(38) $$\begin{array}{c} |{\tilde{\epsilon }}_{r}\left(s\right)|=\frac{4c^{2}{\left|\bar{r}-{\bar{r}}^{\prime }\right|}^{2}}{f_{0}^{\prime 2}}\left|\tilde{\gamma }\left(s\right)\right|+1 \end{array}$$

### 3.6. Capon Method

The Capon algorithm has been used to detect the forest-height profile from a power spectrum [14]. It is implemented by using the same filter as derived in (27)–(33),

(39) $$\begin{array}{ccc} & & \;{\bar{h}}_{C}\left(s\right)=\frac{{\left(\bar{\bar{R}}+\lambda \bar{\bar{I}}\right)}^{-1}\cdot \bar{a}\left(s\right)}{{\bar{a}}^{†}\left(s\right)\cdot {\left(\bar{\bar{R}}+\lambda \bar{\bar{I}}\right)}^{-1}\cdot \bar{a}\left(s\right)} \end{array}$$

The power spectrum is obtained by substituting (39) into (29) to give [14]

(40) $$\begin{array}{ccc} & & \;{\bar{u}}_{C}\left(s\right)={\bar{h}}_{C}^{†}\left(s\right)\cdot \bar{\bar{R}}\cdot {\bar{h}}_{C}\left(s\right)=\frac{1}{{\bar{a}}^{†}\left(s\right)\cdot {\left(\bar{\bar{R}}+\lambda \bar{\bar{I}}\right)}^{-1}\cdot \bar{a}\left(s\right)} \end{array}$$

## 4. Simulations and Discussion

**Figure 5:**
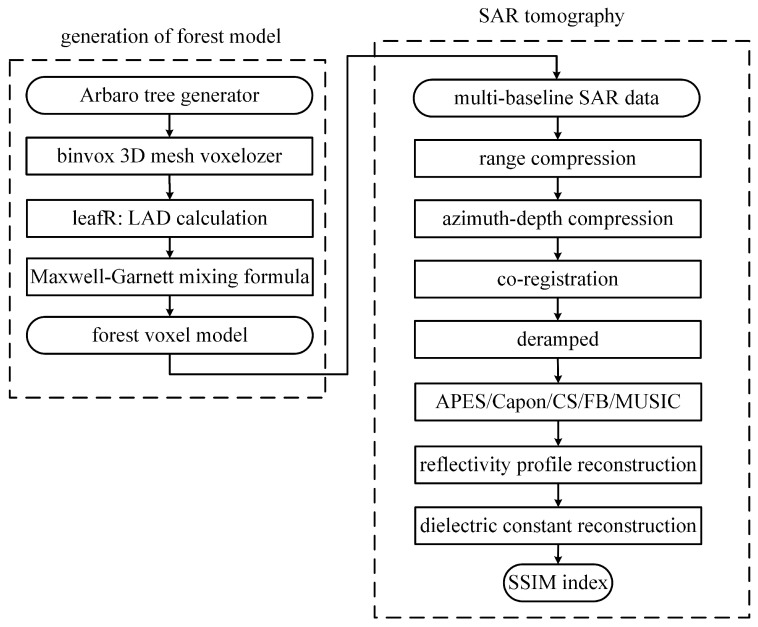
Flowchart of the proposed TomoSAR procedure, including generation of forest model, SAR tomography and validation of results.

Figure 5 shows the flowchart of the proposed TomoSAR procedure, including generation of the forest model, SAR tomography and validation of results.

### 4.1. Parameters for TomoSAR Simulations

Table 1 lists the parameters used in the simulations. The carrier frequency is 1.25 GHz, at which the backscattered signal power was claimed to be more uniform in the elevation (*s*) direction [12]. UAVs have been used to carry SAR, with a typical velocity of 0.1 m/s [32] to 5 m/s [42], moving at a height of 10 m [33] to 200 m [43]. In the simulations, we assume the UAVs fly at a height of H=150 m, with a velocity of $v_{p}=5$ m/s, and the look angle is $\theta_{\ell }=50^{\circ }$. A range bandwidth of 500 MHz [32] or 600 MHz [42] has been reported in UAV-based SAR. The range bandwidth of 300 MHz is chosen to achieve the range resolution of 0.5 m. A pulse repetition frequency of 50 Hz [44] to 1 kHz [33] has been reported. By setting $F_{a}=50$ Hz, the synthetic aperture length is $L_{a}=51.2$ m and the azimuth resolution is Δy=0.54 m. A pulse duration of 10 μs [44] to 100 μs [33] has been used. By setting the pulse duration to $T_{r}=1$ μs, the corresponding range chirp rate is $K_{r}=300$ THz/s.

The elevation resolution is given by $\Delta s=\lambda R_{0}/\left(2B_{n}\right)$. By specifying Δs=0.5 m, compatible with the range resolution and azimuth resolution, the baseline aperture is determined as $B_{n}=57.22$ m. The maximum canopy height in Arbaro is $H_{c}=22$ m [27], which corresponds to the maximum elevation of $H_{c}/\sin\theta_{\ell }=28.7$ m. The elevation ambiguity is given by $s_{\mathrm{amb}}=\lambda R_{0}/\left(2d_{b}\right)$, which must be larger than $H_{c}\left(s\right)$ to avoid elevation ambiguity. By setting $s_{\mathrm{amb}}=28.7$ m, the baseline spacing is $d_{b}=0.49$ m. Finally, the number of baselines is determined from $B_{n}$ and $d_{b}$ as $N_{p}=115$.

The effects of noise are also simulated under the signal-to-noise ratio (SNR) of 0 to −10 dB, where $\mathrm{SNR}=10\log(U_{s}^{2}/U_{n}^{2})$, and $U_{s}$ and $U_{n}$ are the average amplitudes of signal and noise, respectively.

Table 2 lists the parameters of a healthy taramack, a healthy quaking aspen and a dry (dead) tamarack, respectively, to be used in the simulations. The wood moisture and dielectric constant of a deadwood trunk are different from their healthy counterparts. Lower wood moisture implies a lower dielectric constant.

A voxel model of the forest with a resolution of 0.25 m was generated by applying an open-source Binvox [45] on the airborne laser scanning (ALS) point clouds [30]. In this work, the Arbaro [27] is used to generate a 3D tree model, composed of trunk, branches and leaves. The data of the tree model are then mapped to point clouds to form a fine-resolution voxel model by using an open-source CloudCompare software v2.12.4 [46].

The dielectric constants of tree parts can be estimated with empirical formulas [7,47,48,49,50]. The effective dielectric constant of each voxel can be determined by applying proper mixing formulas to the constituent matters within the voxel. A trunk with a diameter larger than the voxel size is modeled as a bunch of voxels filled with trunk matter. The dielectric constant of a voxel composed of leaves and air is estimated by using the Maxwell-Garnett mixing formula [51]. The dielectric constant of a voxel composed of air and branches is estimated by using the Maxwell-Garnett mixing formula for randomly oriented, highly prolate and rotation-symmetric ellipsoidal inclusions [52,53,54].

**Figure 6:**
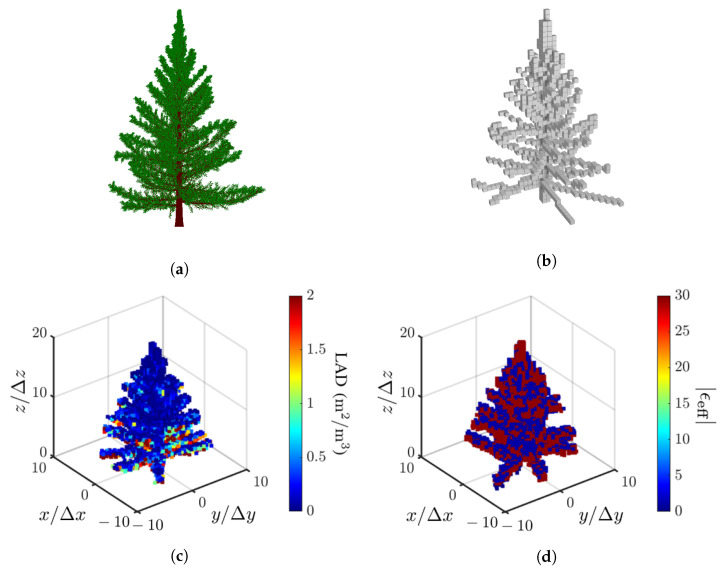
Tree models, (**a**) point clouds generated with Arbaro [27], (**b**) voxel model mapped with Binvox [45], (**c**) LAD model of leaves generated with leafR [55], (**d**) distribution of dielectric constant in voxels.

Figure 6a shows an example of point clouds of a tree generated with Arbaro [27], and Figure 6b shows the voxel model mapped from the point clouds with Binvox [45]. Each voxel is a cubic of size 0.5 m, the same as the range and azimuth resolutions specified in Table 1. The effective dielectric constant in each voxel is estimated by applying the Maxwell-Garnett mixing formula to the constituent leaves, branches and trunks. Figure 6c shows the LAD model of leaves generated with leafR [55], and Figure 6d shows the distribution of the dielectric constant in voxels of the tree model.

### 4.2. Simulations on Virtual Forest

**Figure 7:**
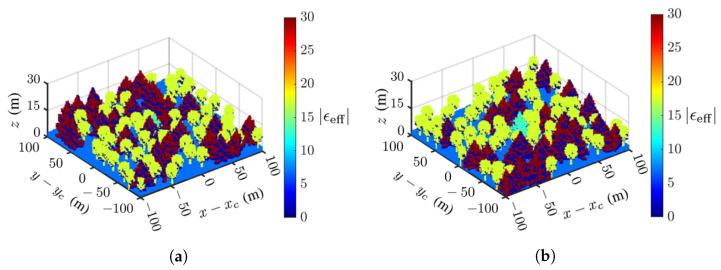
Dielectric-constant profile of virtual forest [27], (**a**) deadwood taramack at center, surrounded by healthy quaking aspen and taramack, (**b**) same as (**a**) except the deadwood taramack is farther away from the surrounding trees.

Inspired by the tamarack-ignited wildfire that burned 68,637 acres in 2021 [56], Figure 7 shows the dielectric-constant profile of two virtual forests, composed of tamaracks and quaking aspen, generated with Arbaro [27]. There are 85 trees randomly dispersed in an area of 100 m × 100 m, with a tree spacing of 2.2–5 m [57]. Figure 7a shows a scenario with a deadwood tamarack at the center, close to the surrounding trees. Figure 7b shows another scenario with the deadwood at the center, but farther away from the surrounding trees. The proposed TomoSAR procedure is aimed to pinpoint this deadwood from the acquired dielectric-constant profile of the forest.

The fidelity of an acquired image *a* against the ground-truth image *b* can be evaluated with a structural similarity index (SSIM) defined as [58]

(41) $$\begin{array}{ccc} & & \;\mathrm{SSIM}\left(a,b\right)=\left(\frac{2\mu_{a}\mu_{b}+c_{1}}{\mu_{a}^{2}+\mu_{b}^{2}+c_{1}}\right)\left(\frac{2\sigma_{ab}+c_{2}}{\sigma_{a}^{2}+\sigma_{b}^{2}+c_{2}}\right) \end{array}$$

where $\mu_{p}$ and $\sigma_{p}$ are the mean and standard deviation, respectively, of image *p*, with p=a,b; $\sigma_{ab}$ is the covariance between images *a* and *b*; and $c_{1}$ and $c_{2}$ are stability constants. The SSIM index lies between 0 and 1; a higher index means the two images are more similar to each other. In the simulations, each image pixel is stored in 8 bits, corresponding to an integer between 0 and L=255. The stability constants are set to $c_{1}={\left(0.01L\right)}^{2}$ and $c_{2}=\left(0.03L^{2}\right)$.

The fidelity of an acquired image *a* against the ground-truth image *b* can also be evaluated with a root-mean-square error (RMSE) defined as [59]

(42) $$\begin{array}{ccc} & & \;\mathrm{RMSE}\left(a,b\right)=\sqrt{\frac{1}{P}\sum_{p=1}^{P}{\left(a_{p}-b_{p}\right)}^{2}} \end{array}$$

where $a_{p}$ and $b_{p}$ are the values of the *p*th pixel in images *a* and *b*, respectively, and *P* is the number of pixels in each image.

**Figure 8:**
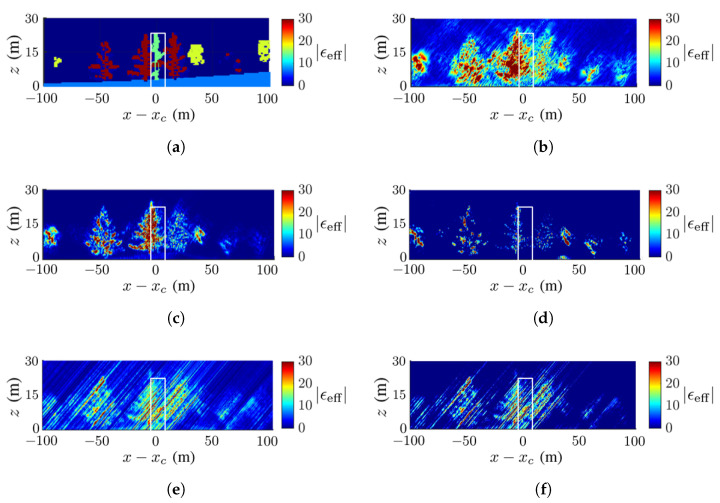
Dielectric-constant profile at $y=y_{c}$ in Figure 7a, deadwood tamarack is enclosed within a white square, (**a**) ground truth, (**b**) CS, (**c**) FB, (**d**) MUSIC, (**e**) APES, (**f**) Capon.

Figure 8 shows the ground-truth dielectric-constant profile in Figure 7a at $y=y_{c}$ and the reconstructed images with the methods of CS, FB, MUSIC, APES and Capon, respectively. Around $x=x_{c}$ of the reconstructed images, the deadwood is slightly obscured by the surrounding healthy quaking aspen and taramack, which have a higher dielectric constant than the deadwood due to the difference in moisture content.

Figure 8c,d show a clear background, while Figure 8b,e,f manifest artifact streaks in the background. The streaks in the acquired images have similar patterns but different magnitudes, which may be attributed to numerical errors in the covariance matrix.

Figure 8b,c restore the tree shape and dielectric constant more accurately than the other three reconstructed images. The contrast of the dielectric constant in the canopy is less obvious in Figure 8b than in Figure 8c.

The SSIM index of each image against the ground-truth image is listed in Table 3, which is consistent with the visual inspection. The ground is blurred and only partially revealed, possibly because the incident wave and backscattered wave are attenuated by the canopies. Since the deadwood is very close to the surrounding healthy trees in this scenario, the former is not well discernible in all the reconstructed images.

**Figure 9:**
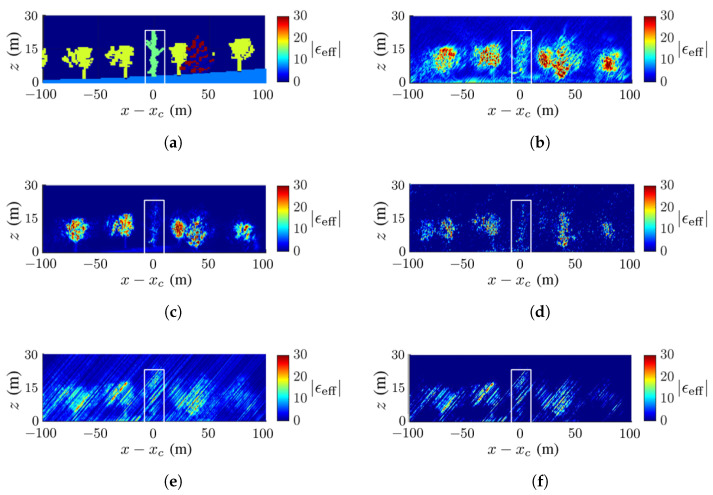
Dielectric-constant profile at $y=y_{c}$ in Figure 7b, deadwood tamarack is enclosed within a white square, (**a**) ground truth, (**b**) CS, (**c**) FB, (**d**) MUSIC, (**e**) APES, (**f**) Capon.

Figure 9 shows the ground-truth dielectric-constant profile in Figure 7b at $y=y_{c}$ and the reconstructed images with the methods of CS, FB, MUSIC, APES and Capon, respectively. In this scenario, the deadwood is farther away from the surrounding healthy trees than in Figure 7a. In general, Figure 9b,c match the ground-truth image in Figure 9a better than the other three images. Figure 9b shows a higher contrast of the dielectric constant than the other reconstructed images, and Figure 9c shows the sharpest tree shape and the cleanest background compared with the others.

Figure 9b,e,f manifest similar patterns of artifact streaks, which may be related to numerical errors in the covariance matrix of SLC images. Figure 9d displays some speckles in the background, which are not observed in its counterpart of Figure 8d. The deadwood part in Figure 9b,c matches well to that in the ground-truth image.

No calibration is needed to acquire the image in Figure 9b with the CS method, but some artifact streaks appear on the background. The image in Figure 9c acquired with the FB method vividly imitates the true forest and the background is clean.

The proposed approach can be used to reconstruct the 3D dielectric-constant profile of a target region at a fine spatial resolution by deploying a swarm of UAVs as platforms. The time decorrelation issue of multi-pass operations with airplanes or satellites is also avoided.

### 4.3. Effects of Noise

The fidelity of the acquired TomoSAR images may be deteriorated by noises from the atmosphere and other sources, which is quantified against the backscattered signal in terms of the signal-to-noise ratio (SNR). Next, simulations on the two scenarios in Figure 7a,b are conducted at SNR=0 dB, −5 dB and −10 dB, respectively.

**Figure 10:**
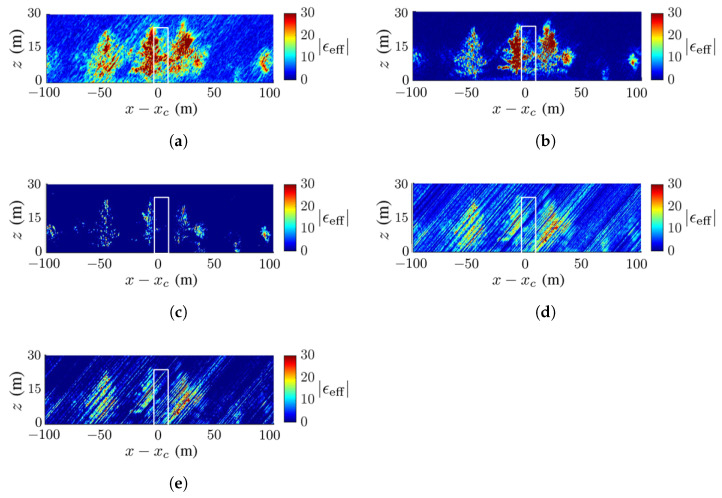
Dielectric-constant profile at $y=y_{c}$ in Figure 7a, deadwood tamarack is enclosed within a white square, SNR=0 dB, (**a**) CS, (**b**) FB, (**c**) MUSIC, (**d**) APES, (**e**) Capon.

Figure 10 shows the TomoSAR images acquired on the scenario in Figure 7a under SNR=0 dB. Compared with their counterparts in Figure 8, the images in Figure 10a with the CS method and Figure 10d with the APES method look blurrier, while the images in Figure 10b,c,e are barely affected, consistent with the SSIM indices listed in Table 3. The SSIM index suggests that the acquired image with the MUSIC method matches the best to the ground-truth image.

**Figure 11:**
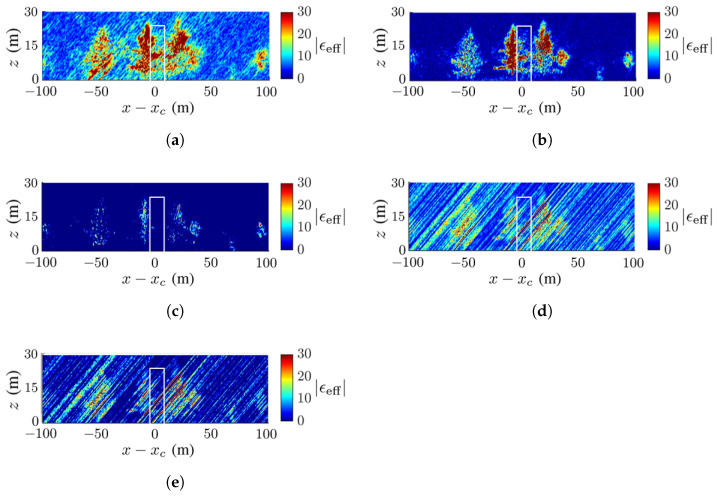
Dielectric-constant profile at $y=y_{c}$ in Figure 7a, deadwood tamarack is enclosed within a white square, SNR=−5 dB, (**a**) CS, (**b**) FB, (**c**) MUSIC, (**d**) APES, (**e**) Capon.

**Figure 12:**
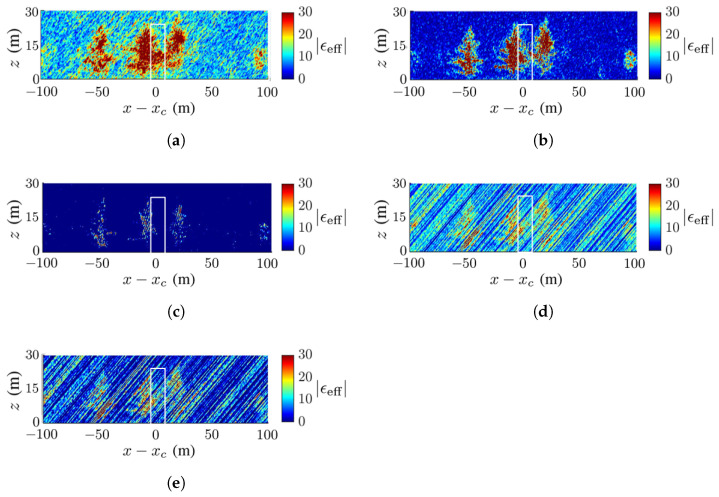
Dielectric-constant profile at $y=y_{c}$ in Figure 7a, deadwood tamarack is enclosed within a white square, SNR=−10 dB, (**a**) CS, (**b**) FB, (**c**) MUSIC, (**d**) APES, (**e**) Capon.

Figure 11 and Figure 12 show the TomoSAR images acquired for the scenario in Figure 7a under SNR=−5 dB and SNR=−10 dB, respectively. The FB and the MUSIC methods outperform the other three methods in terms of forest configuration and background cleanliness. The artifact streaks in the images with the APES and Capon methods are strong enough to engulf the trees, especially under SNR=−10 dB. The performance of these two methods relies on the accurate estimation of the covariance matrix from the SLC images. Strong speckles appear in the images acquired with the CS method, but the forest configurations are still recognizable. Among these five methods, the MUSIC method acquires the cleanest background and the FB method best preserves the forest configuration.

**Figure 13:**
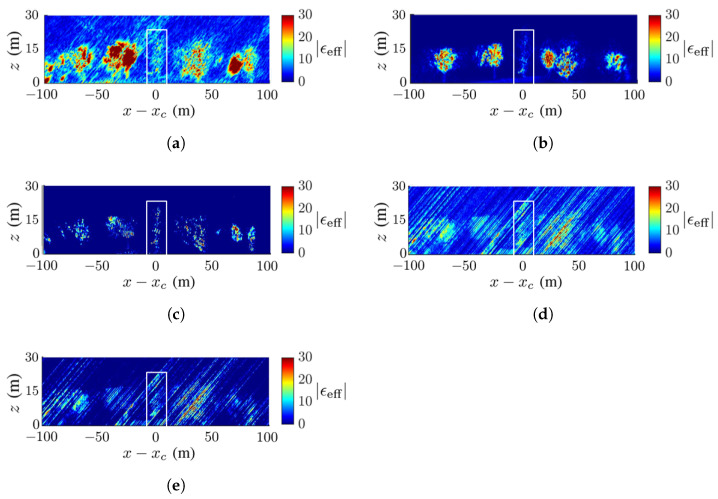
Dielectric-constant profile at $y=y_{c}$ in Figure 7b, deadwood tamarack is enclosed within a white square, SNR=0 dB, (**a**) CS, (**b**) FB, (**c**) MUSIC, (**d**) APES, (**e**) Capon.

Figure 13 shows the TomoSAR images acquired for the scenario in Figure 7b under SNR=0 dB. In this scenario, the deadwood is slightly separated from the surrounding trees. The image acquired with the CS method manifests the highest contrast of dielectric constants compared with the other methods. Compared with its counterpart in Figure 9b, background speckles grow stronger. The background in the images acquired with the FB and MUSIC methods are almost unaffected by noise. The forest configuration is also well preserved with these two methods. The deadwood in the center is much better reconstructed in the images as compared to their counterparts acquired for the scenario in Figure 7a. The artifact streaks grow strong in the images acquired with the APES and Capon methods, respectively, similar to their counterparts in Figure 10.

**Figure 14:**
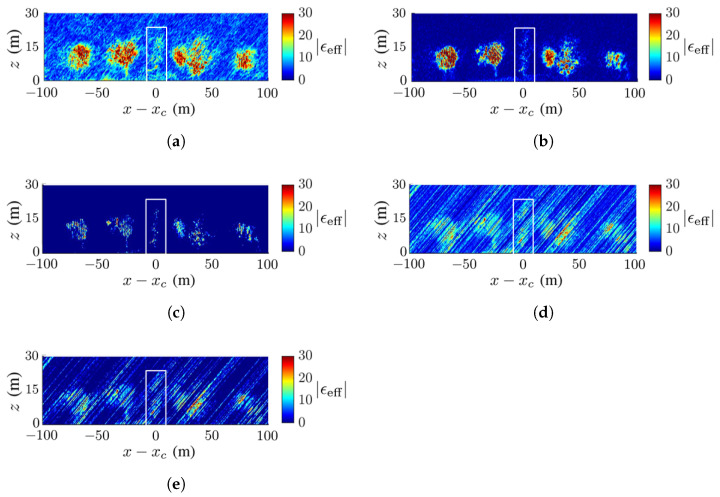
Dielectric-constant profile at $y=y_{c}$ in Figure 7b, deadwood tamarack is enclosed within a white square, SNR=−5 dB, (**a**) CS, (**b**) FB, (**c**) MUSIC, (**d**) APES, (**e**) Capon.

**Figure 15:**
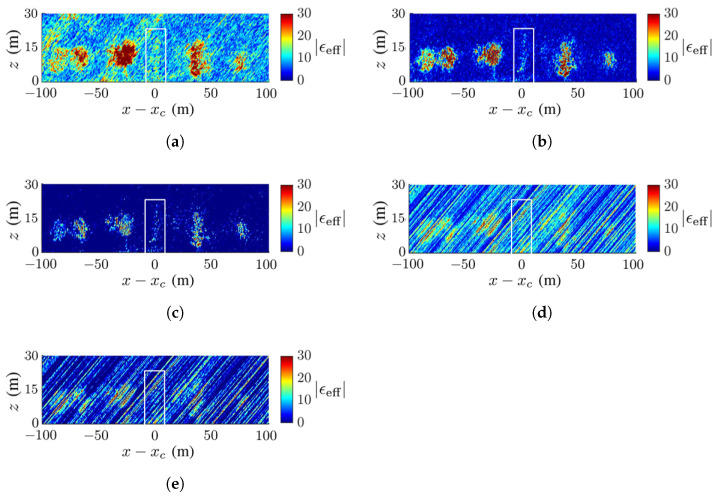
Dielectric-constant profile at $y=y_{c}$ in Figure 7b, deadwood tamarack is enclosed within a white square, SNR=−10 dB, (**a**) CS, (**b**) FB, (**c**) MUSIC, (**d**) APES, (**e**) Capon.

Figure 14 and Figure 15 show the TomoSAR images acquired for the scenario in Figure 7b under SNR=−5 dB and SNR=−10 dB, respectively. As the noise increases, speckles grow stronger in the images acquired with the CS method, speckles grow slightly with the FB and MUSIC methods, and artifact streaks grow strong with the APES and Capon methods. Under SNR=−10 dB, the trees are barely recognizable with the APES and Capon methods. The deadwood is discernible with the CS, FB and MUSIC methods, even under SNR=−10 dB. The FB method delivers the highest contrast of dielectric constant in the image, while the MUSIC method delivers the highest SSIM index under noisy conditions.

Table 3 lists the SSIM index of the images in Figure 8, Figure 9, Figure 10, Figure 11, Figure 12, Figure 13, Figure 14 and Figure 15. In general, the FB method achieves the highest SSIM index under noise-free conditions, and the MUSIC method achieves the highest SSIM index under noisy conditions.

Table 4 lists the RMSE indices of the images in Figure 8, Figure 9, Figure 10, Figure 11, Figure 12, Figure 13, Figure 14 and Figure 15. As the noise level is raised, the Capon method and the APES method tend to generate more stripes in the reconstructed images; hence, their RMSE indices are relatively higher than the other methods. The FB and MUSIC methods yield relatively lower RMSE indices, and the reconstructed images better resemble the ground-truth images. In general, the MUSIC method or the CS method yields the lowest RMSE index across the whole area, while the FB method yields the lowest RMSE index within the square area.

### 4.4. Performance Comparison of Imaging Methods

In general, the FB method and the MUSIC method deliver better TomoSAR images of the simulated forest scenarios than the other three imaging methods. The compressive-sensing (CS) method has the flexibility of reconstructing TomoSAR images with sparse baselines and does not require normalization, but it takes more computational loading than the other methods.

The hyperparameter K in the MUSIC method is sensitive to the number of phase centers, which can be empirically adjusted to enhance the acquired images under different noise conditions. Under noise-free conditions, the FB method reconstructs clear images and is less prone to developing ghost targets. However, it may not effectively identify low dielectric-constant areas containing deadwood. Under severe noise, the FB method may render a noisy background image. The FB method takes the lowest computational load among these methods.

The Capon method and the APES method follow the same process of deriving the optimal filters. Both methods tend to produce stripes along the elevation direction in the images. The APES method was originally tried to avoid normalization, but it did not work out as expected.

The Capon and the FB methods proved to be the most computationally efficient. The quality of reconstructed images with the FB method excels under noise-free conditions, and that with the MUSIC method excels under noisy conditions.

The CS method does not require normalization of the reflectivity function as the other four methods do. Future work may be developed to reduce the computational load of the CS method and remove the constraint of normalization on the FB, Capon, APES and MUSIC methods.

### 4.5. Highlighted Contributions

In conventional TomoSAR imaging methods, the backscattered signals received along multiple tracks are first compressed and co-registered along a specific elevation line segment in the target volume by using an empirical point spread function. Then, an inverse algorithm is applied to reconstruct the image along the elevation line segment. However, the dielectric-constant profile in the target volume cannot be accurately estimated without calibration or normalization. In our TomoSAR procedure, the backscattered signals are related to the reflectivity function via the scattering theory, and the image at specific azimuth *y* and specific depth $r^{\prime \prime }$ is reconstructed via a focusing function $\xi_{nk}$, without resorting to calibration or normalization. Five different inverse methods are used to acquire the reflectivity function along the specified elevation line segment. The acquired images are analyzed to validate the proposed procedure and compare the performance of these five inverse methods in TomoSAR imaging.

The novelties and contributions of this work are summarized as follows.

1. A complete and rigorous TomoSAR imaging procedure is developed by integrating scattering theory and signal processing models to acquire a 3D dielectric-constant profile of a forest at decimeter resolution. A focusing function is derived to upgrade the point spread function adopted in conventional TomoSAR methods, without resorting to empirical assumption or calibration.
2. The imaging task can be carried out in near real time with a swarm of UAVs to scout hot-spots of wildfire hazards.
3. A vivid forest voxel model is developed by integrating the Arbaro tree generator, Maxwell-Garnett mixing formulas and leaf area index (LAD) for calculating the dielectric constant of a leaf, branch and trunk.

## 5. Conclusions

A complete and rigorous TomoSAR imaging procedure is developed by integrating scattering theory and signal processing models to acquire a dielectric-constant profile of a forest at decimeter resolution. The imaging task can be carried out with a swarm of UAVs to scout hot-spots of wildfire hazards in a forest. A vivid forest voxel model is developed by integrating the Arbaro tree generator, Maxwell-Garnett mixing formulas and leaf area index (LAD) for calculating the dielectric constant of a leaf, branch and trunk. Simulation results validate the efficacy of the proposed procedure. Five different inverse methods are used to acquire and compare the dielectric-constant profiles in two forest scenarios under three different signal-to-noise ratios.

The simulation results indicate that a deadwood close to the surrounding trees is difficult to pinpoint. Future efforts are needed to enhance the spatial resolution and the contrast of the reflectivity function in the acquired images. The five imaging methods are primarily used to compare their merits and shortcomings on the proposed scenario. Future efforts are needed to update or tailor these methods for better TomoSAR imaging on forests.

## Figures and Tables

**Table 1:** Parameters for TomoSAR simulations.

Parameter	Symbol	Value
carrier frequency	$f_{c}$	1.25 GHz
range bandwidth	$B_{r}$	300 MHz
pulse duration	$T_{r}$	1 μs
range chirp rate	$K_{r}$	300 THz/s
range sampling rate	$F_{r}$	512 MHz
pulse repetition frequency	$F_{a}$	50 Hz
range samples	$N_{r}$	512
azimuth samples	$N_{a}$	512
platform height	*H*	150 m
platform velocity	$v_{p}$	5 m/s
look angle	$\theta_{\ell }$	50${}^{\circ }$
closest slant range	$R_{0}$	233.4 m
synthetic aperture length	$L_{a}$	51.2 m
slant range resolution	Δr	0.5 m
azimuth resolution	Δy	0.54 m
elevation resolution	Δs	0.5 m
baseline aperture	$B_{n}$	57.22 m
elevation ambiguity	$S_{\mathrm{amb}}$	28.72 m
baseline spacing	$d_{b}$	0.49 m
number of baselines	$N_{p}$	115
maximum canopy height	$H_{c}$	22 m

**Table 2:** Parameters of forest model.

Parameter	Symbol	Value	Value	Value
tree species		tamarack	quaking aspen	tamarack (dry)
country		U.S.	U.S.	U.S.
canopy height	$H_{c}$	21 m	15 m	21 m
trunk diameter	$D_{t}$	33 cm	25 cm	33 cm
DC of trunk	$\epsilon_{w}$	29.47−j9.39	17.48−j5.92	8.27−j2.83
wood moisture const.	$M_{g}$	0.7 g/m^3^	0.5 g/m^3^	0.2 g/m^3^
radius of branch	$r_{b}$	0.03 m	0.03 m	0.03 m
length of branch	$\ell_{b}$	0.5 m	0.5 m	0.5 m
density of branch	$n_{b}$	20/m^3^	20/m^3^	20/m^3^
volume fraction of branches	$V_{b}$	0.23	0.23	0.23
DC of leaf	$\epsilon_{\ell }$	15.33−j5.26	13−j6.38	no leaf
avg. leaf thickness	$t_{\ell }$	0.03 cm	0.03 cm	no leaf
leaf area index	LAI	[0,5.78]	[0,2.59]	[0,2.59]
volume fraction of leaves	$V_{\ell }$	[0,0.0173]	[0,0.0132]	[0,0.0132]
effective DC of air&leaf	$\epsilon_{a\ell }$	$\epsilon_{a\ell }^{\prime }:\left[1,1.13\right]$, $\epsilon_{a\ell }^{\prime \prime }:\left[0,0.06\right]$	$\epsilon_{a\ell }^{\prime }:\left[1,1.13\right]$, $\epsilon_{a\ell }^{\prime \prime }:\left[0,0.05\right]$	$\epsilon_{a\ell }^{\prime }:\left[1,1.13\right]$, $\epsilon_{a\ell }^{\prime \prime }:\left[0,0.05\right]$
effective DC of air&leaf&branch	$\epsilon_{\mathrm{eff}}$	$\epsilon_{\mathrm{eff}}^{\prime }:\left[1,29.49\right]$	$\epsilon_{\mathrm{eff}}^{\prime }:\left[1,17.48\right]$	$\epsilon_{\mathrm{eff}}^{\prime }:\left[1,8.27\right]$
DC of ground	$\epsilon_{g}$	4	4	4
voxel size	$h^{3}$	0.5${}^{3}$ m^3^	0.5${}^{3}$ m^3^	0.5${}^{3}$ m^3^

DC: dielectric constant.

**Table 3:** SSIM indices of acquired images.

Figure 8	CS	FB	MUSIC	APES	Capon
forest (whole area)	0.629	0.701	0.613	0.556	0.491
deadwood (square)	0.193	0.333	0.300	0.234	0.305
**Figure 9**	CS	FB	MUSIC	APES	Capon
forest (whole area)	0.683	0.704	0.635	0.568	0.572
deadwood (square)	0.430	0.454	0.487	0.416	0.456
**Figure 10**, SNR =0 dB	CS	FB	MUSIC	APES	Capon
forest (whole area)	0.601	0.703	0.723	0.484	0.549
deadwood (square)	0.216	0.294	0.454	0.359	0.419
**Figure 11**, SNR =−5 dB	CS	FB	MUSIC	APES	Capon
forest (whole area)	0.525	0.688	0.730	0.406	0.561
deadwood (square)	0.208	0.275	0.454	0.324	0.389
**Figure 12**, SNR =−10 dB	CS	FB	MUSIC	APES	Capon
forest (whole area)	0.371	0.597	0.731	0.322	0.461
deadwood (square)	0.162	0.182	0.453	0.268	0.312
**Figure 13**, SNR =0 dB	CS	FB	MUSIC	APES	Capon
forest (whole area)	0.582	0.666	0.677	0.477	0.528
deadwood (square)	0.427	0.448	0.485	0.403	0.460
**Figure 14**, SNR =−5 dB	CS	FB	MUSIC	APES	Capon
forest (whole area)	0.600	0.691	0.690	0.470	0.618
deadwood (square)	0.422	0.442	0.485	0.391	0.439
**Figure 15**, SNR =−10 dB	CS	FB	MUSIC	APES	Capon
forest (whole area)	0.417	0.608	0.679	0.304	0.498
deadwood (square)	0.350	0.413	0.480	0.277	0.344

**Table 4:** RMSE indices of acquired images.

Figure 8	CS	FB	MUSIC	APES	Capon
forest (whole area)	**0.95**	**0.95**	1.18	1.39	1.23
deadwood (square)	0.68	1.33	**0.66**	0.94	1.60
**Figure 9**	CS	FB	MUSIC	APES	Capon
forest(whole area)	1.06	**0.85**	0.86	1.6	1.15
deadwood(square)	**0.83**	1.1	1.16	1.21	1.27
**Figure 10**, SNR =0 dB	CS	FB	MUSIC	APES	Capon
forest(whole area)	3.6	2.79	**2.37**	3.84	3.69
deadwood(square)	2.91	3.66	**2.88**	3.06	3.12
**Figure 11**, SNR =−5 dB	CS	FB	MUSIC	APES	Capon
forest(whole area)	4.66	**2.38**	3.08	4.13	3.54
deadwood(square)	**3.19**	**3.19**	4.48	4.06	3.26
**Figure 12**, SNR =−10 dB	CS	FB	MUSIC	APES	Capon
forest(whole area)	4.36	4.72	**3.6**	6.24	5.00
deadwood(square)	3.16	**3.08**	5.16	6.04	5.48
**Figure 13**, SNR =0 dB	CS	FB	MUSIC	APES	Capon
forest(whole area)	2.76	3.12	**2.25**	3.51	4.74
deadwood(square)	2.64	**2.07**	3.24	3.66	3.66
forest(whole area)	**2.87**	4.55	3.05	3.19	3.33
deadwood(square)	4.45	**2.66**	4.59	4.40	3.99
**Figure 15**, SNR =−10 dB	CS	FB	MUSIC	APES	Capon
forest(whole area)	**3.28**	4.16	4.44	4.68	6.32
deadwood(square)	3.52	**2.76**	3.76	5.04	4.56

## Data Availability

Publicly available datasets were analyzed in this study.
